# Assessment of abdominal symptoms and intestinal inflammation in children and adolescents with cystic fibrosis without highly effective modulator therapy^✰^

**DOI:** 10.1016/j.jped.2026.101552

**Published:** 2026-05-09

**Authors:** Gabriel Cezar dos Santos, Lorenna Cristina Montera, Lívia Moreira Genaro, Beatriz Alves Guerra Rodrigues, Raquel Franco Leal, Antonio Fernando Ribeiro, Jose Dirceu Ribeiro, Elizete Aparecida Lomazi

**Affiliations:** a Universidade Estadual de Campinas (UNICAMP), Faculdade de Ciências Médicas (FCM), Graduate Program in Child and Adolescent Health, Campinas, SP, Brazil; b Universidade Estadual de Campinas (UNICAMP), Faculdade de Ciências Médicas (FCM), Surgery Department, Gastrocenter, Colorectal Surgery Unit, Inflammatory Bowel Disease Research Laboratory (LABDII), Campinas, SP, Brazil; c Universidade Estadual de Campinas (UNICAMP), Faculdade de Ciências Médicas (FCM), Department of Pediatrics, Campinas, SP, Brazil

**Keywords:** Cystic fibrosis, Abdominal pain, Inflammation, Intestinal, Fecal calprotectin

## Abstract

**Objective:**

Cystic fibrosis (CF) is a rare genetic disease caused by mutations in the CFTR gene, resulting in a dysfunctional protein that affects various systems in the body, including the gastrointestinal (GI) tract. The changes caused by CF in the GI tract include dysmotility, dysbiosis, intestinal inflammation, and abdominal symptoms such as pain, altered bowel habits, and distension, which are frequent in people with CF. This study aims to establish the prevalence of these complaints in children with CF and investigate associations between symptoms, clinical conditions, and markers of intestinal inflammation.

**Methods:**

Twenty-two pediatric patients with a genetic diagnosis of CF, followed at a Brazilian reference center, were included. Methods involved a systematic assessment of gastrointestinal symptoms using a questionnaire, biochemical analysis of fecal calprotectin (FC) as a marker of intestinal inflammation, and collection of clinical, anthropometric, laboratory, and imaging data from medical records.

**Results:**

86.3% (*n* = 19) of participants showed elevated FC levels (> 50 gt; 50 mcg/g), indicating a high prevalence of intestinal inflammation. The most common abdominal symptoms were pain and flatulence, present in about half of the patients. No significant associations were observed between FC levels and clinical or symptomatic variables.

**Conclusion:**

There is a high prevalence of intestinal inflammation and abdominal symptoms in this sample of Brazilian children with CF, highlighting the importance of systematically investigating these symptoms.

## Introduction

Cystic fibrosis (CF) is a rare autosomal recessive disease whose etiology is due to mutations in the CFTR gene, leading to the transcription of a dysfunctional CFTR protein, which is the source of the clinical manifestations of the disease. The CFTR protein transports chloride and bicarbonate in secretory epithelia. It is most notably present in the airways, sweat glands, gastrointestinal tract (GI), pancreas, and vas deferens [1]. In the lungs, the production of abnormal secretions due to transporter dysfunction results in the formation of thick mucus, which reduces the effectiveness of clearance defense mechanisms and leads to progressive inflammatory and fibrotic damage [2]. In the pancreas, the thicker secretion causes successive obstructions in the pancreatic ducts, inflammation, and progressive tissue damage [1]. In the biliary tract, a similar process occurs, with thick bile secretion, cholestasis, and hepatic tissue damage [3].

There are also typical intestinal changes resulting from CF, associated with the involvement of its accessory organs. Evidence shows that various mechanisms triggered by CFTR mutation lead to alterations in the normal functioning of the GI tract, such as esophageal, gastric, and intestinal [4,5] dysmotility; increased proliferation of intestinal microorganisms associated with dysbiosis; and chemical modifications of the intestinal contents, such as lower pH, undigested nutrients, and metabolites produced by the gut microbiota [6].

All these manifestations clinically present as conditions typically associated with CF, including exocrine pancreatic insufficiency, CF-related liver disease, gastroesophageal reflux disease, distal intestinal obstruction syndrome, meconium ileus, cholelithiasis, and chronic constipation, among others [7].

Given this myriad of CF manifestations in the GI tract, abdominal symptoms such as abdominal pain, altered bowel habits, abdominal distension, and pain during defecation, among others, are quite common in people with CF [8]. Intestinal inflammation is a feature that can be associated both with the pathophysiological changes generated by the CFTR protein mutation and with the most common abdominal symptoms. Several studies have found endoscopic, histological, and biochemical evidence of inflammation in the intestines of people with CF and have proposed biological mechanisms to justify such inflammation [9].

Fecal calprotectin (FC) is a zinc- and calcium-binding protein found in the cytoplasm of macrophages and activated granulocytes and secreted by epithelial cells exposed to inflammatory cytokines or bacterial products, playing a significant role in the acute phase of inflammation [10]. It is found in the stool due to the migration of neutrophils to the intestines in response to the presence of pathogens and in other inflammatory conditions not mediated by microorganisms. As it is resistant to degradation by intestinal proteases, it remains stable in the intestinal lumen and in defecated feces. Although it is already established as a reliable method for assessing inflammation in inflammatory bowel disease, its role in CF is not yet fully elucidated. Lazarotto et al., in the most recent systematic review on the subject, highlight that research limitations such as small sample sizes or short follow-up periods, as well as the fact that CF is a multisystemic condition, make it more challenging to confirm that FC consistently and specifically reflects the level of intestinal inflammation in people with CF [11], reinforcing the importance of further studies on this marker in CF.

By systematically assessing abdominal complaints, biochemical analysis of the intestinal inflammation marker FC, and other common conditions in people with CF, this study aims to establish the prevalence of abdominal complaints in children with CF and investigate whether there is an association between abdominal symptoms, clinical conditions, and FC levels.

## Methods

### Study design

Case series, with mixed (prospective and retrospective) observational data collection. The study participants were selected among CF patients followed at a Brazilian reference center. The center treats children and adolescents referred after diagnostic suspicion, usually identified by neonatal screening. In 2024, care was provided to 256 patients with confirmed or suspected CF diagnoses, of whom 174 had genetic testing showing the F508del variant in homozygosity or heterozygosity with another pathogenic variant. All patients from this clinic with a genetic diagnosis of CF (two pathogenic genetic variants, at least one being F508del) were invited to participate. The other patients were not invited due to being ineligible for triple CFTR protein modulator therapy under the current Brazilian national health system (SUS) protocols. A questionnaire on abdominal symptoms and bowel habits was administered to children and adolescents with CF, and a stool sample was collected at the same time. Subsequently, clinical data from the medical records of these participants were gathered. Those who brought inadequate stool samples, had non-CF-related abdominal disease at the time, or did not agree with any condition of the informed consent/assent form were excluded. Data collection was carried out between April 2024 and April 2025. The study was approved by the Research Ethics Committee of the State University of Campinas (UNICAMP).

### Gastrointestinal symptom assessment

Symptoms were systematically assessed in all research participants by the research team at the time of stool sample collection for biomarker analysis of inflammation. The patient responded to questions either independently or with assistance from family members or guardians. The questionnaire was based on a validated instrument for assessing gastrointestinal symptoms and related aspects in individuals with CF, called the CFAbd Score [11]. From this instrument, the authors selected specific questions addressing gastrointestinal symptoms and bowel habits, which were administered to the participants. They were asked whether symptoms (abdominal pain, abdominal distension, flatulence, constipation, and delayed evacuation) had been present during the previous two weeks. Participants also reported the number of bowel movements per day, the intensity and location of abdominal pain (if present), and stool characteristics. Pain intensity was assessed using a visual analog scale ranging from 0 to 10. The location of pain was indicated or marked by the participant on an abdominal diagram. Finally, to evaluate stool appearance, participants were shown the Bristol Stool Scale and asked which drawing(s) best represented the stools passed in the last two weeks. A visual analog scale was used for pain classification and duration. All questions referred to the two weeks preceding the questionnaire.

### Stool sample collection

Participants were asked to bring a stool sample on the day of the medical appointment, from a bowel movement that occurred that day or at most two days before the visit. Participants were instructed to store the collected sample in a dry container and keep it refrigerated at home immediately after collection until it was brought to the healthcare facility. Upon arrival, samples were collected by researchers, identified, and stored at −20 °C. Subsequently, samples underwent biochemical analysis using the BÜHLMANN Quantum Blue® fCAL extended test for quantification of calprotectin in stool, with a detection range of 30–1000 mg/g.

### Medical record data collection

Participants’ medical records were accessed to collect laboratory and imaging data from routine follow-up, anthropometric data, and health conditions associated with CF.

### Data analysis

Data were analyzed descriptively and inferentially using SPSS 25.0 software. For all analyses, a p-value < 0.05 was considered statistically significant. In descriptive analysis of qualitative variables, absolute and relative frequencies were calculated. Associations between nominal qualitative variables were performed using the chi-square test. Comparison of quantitative and ordinal variables between two independent groups used the Mann-Whitney test. Correlation between continuous quantitative variables was assessed using the Spearman rank correlation test.

## Results

Twenty-two children participated in the study. Although many patients were eligible, few provided adequate stool samples. Furthermore, the study was funded to acquire the necessary supplies for analysis of stool samples from up to 22 participants, which was the main factor limiting the sample size. The most relevant characteristics of the sample are described in Table 1.

**Table 1:** Sample characteristics.

Characteristic	n
Number of cases	22
Male sex	13 (59.1%)
Age (mean, IQR)	9.9 (4.25) years
BMI percentile (mean, IQR)	36.6 (43)
Height percentile (mean, IQR)	39.5 (47.4)
FEV1 (median, range)	86.6% (30 - 125)
Mild respiratory disorder	3 (13.6%)
Moderate respiratory disorder	2 (9.1%)
Severe respiratory disorder	1 (4.5%)
Using proton pump inhibitor	0
Chronic antibiotic use	16 (72.6%)
Inhaled antibiotic use	10 (45.4%)
Antibiotic in the last month	3 (13.6%)
Current exacerbation	8 (36.4%)
Colonization by *P. aeruginosa*	9 (40.9%)
Pancreatic enzyme replacement therapy	18 (81.8%)
Chronic laxative use	3 (13.6%)
CF-related liver disease	2 (9.1%)
Cholecystopathy (biliary sludge)	2 (9.1%)
GERD	0
Personal history of meconium ileus	2 (9.1%)

BMI, Body Mass Index; IQR, interquartile range; FEV1, forced expiratory volume in the 1st minute; CFLD, cystic fibrosis–associated liver disease; GERD, gastroesophageal reflux disease.

Only 4 participants (18.2%) had a BMI compatible with thinness (< 3rd percentile), one of whom also had short stature. Another 3 participants (13.6%) had a BMI compatible with overweight (> 85th percentile). All others (68.2%) had anthropometric measurements within the normal range for BMI and height (between 3rd and 85th percentiles for BMI and > 3rd percentile for height).

Regarding chronic antibiotic use, it is worth noting that the clinic follows Brazilian CF treatment guidelines, prescribing azithromycin three times a week to protect lung function and reduce pulmonary exacerbation risk, and recommends inhaled antibiotics (colistimethate sodium or tobramycin) for patients with airways colonized by P. aeruginosa [12]. The described antibiotic therapies were grouped under “chronic antibiotic use” in Table 1.

With respect to pancreatic enzyme replacement therapy (PERT), all participants diagnosed with exocrine pancreatic insufficiency reported consistent use of PERT, and none were taking proton pump inhibitors (PPI). In this clinic, PPI therapy is started to improve the effectiveness of PERT, mainly when the pancreatic enzyme dose is considered adequate, but the patient continues to show clinical or laboratory signs of poor digestion and nutrient absorption, which was not the case for any of the participants.

The two participants with CF-related liver disease had persistently elevated liver enzymes, mainly gamma-glutamyl transferase, and/or abnormalities on abdominal ultrasound following the Williams criteria. Possible etiologies such as drug toxicity, alpha-1 antitrypsin deficiency, Wilson’s disease, autoimmune hepatitis, viral hepatitis, and celiac disease were ruled out. Both study participants were asymptomatic regarding CF-related liver disease.

Although stool samples were collected from 22 participants, 3 brought samples at times when the team responsible for administering questionnaires was unavailable. In these cases, stools were collected, but questionnaires were not administered. Table 2 displays the participants’ questionnaire responses.

**Table 2:** Abdominal symptoms questionnaire responses^a^.

Question	n (%)
Total respondents	19 (100%)
Abdominal pain	8 (42.1%)
Abdominal pain intensity (range 1 to 10)	
1 to 3	2 (10.5%)
4 to 7	3 (15.8%)
8 to 10	3 (15.8%)
Abdominal distension	4 (21%)
Flatulence	11 (57.9%)
Intestinal constipation	3 (15.8%)
Delayed evacuation	4 (21%)
Passage of hard stools at least once	11 (57.9%)
Alternation between soft and hard stools	2 (10.5%)
Average of 4 evacuations per day	2 (10.5%)

a The questionnaire referred to symptoms experienced in the past 2 weeks.

Among the participants who reported abdominal pain, the pain was most frequently located in the middle region of the abdomen, with most complaints centered in the mesogastrium (23%), followed by the right flank (18%), and both the left flank and right iliac fossa (14% each).

**Fig. 1:**
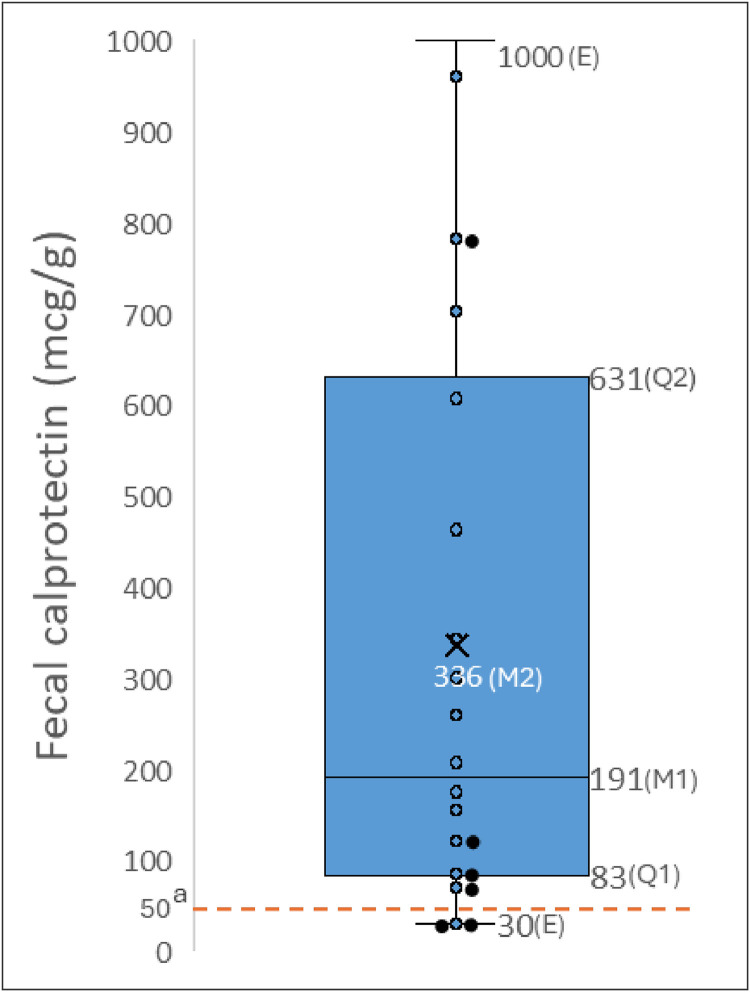
Distribution of CF measurements in the sample^a^. ^a^ The cutoff value for intestinal inflammation is 50 mg/g. E: extreme values; Q3: third quartile; M2: mean; M1: median; Q1: first quartile.

The distribution of the FC values found is described in Figure 1. The cut-off value suggested by the manufacturer, from which intestinal inflammation is considered present, is 50 mg/g. Only 3 participants (13.7%) showed normal levels of FC.

To investigate whether there was an association between the level of intestinal inflammation and the presence of symptoms or other clinical characteristics, the Mann-Whitney test was applied. The results are shown in Table 3. There was no significant correlation between the continuous variables age (p = 0.73, Spearman rank correlation test), BMI percentile (p = 0.41, Spearman rank correlation test), height percentile (p = 0.057, Spearman rank correlation test), and pancreatic enzyme dose (p = 0.08, Spearman rank correlation test) and FC level. The median pancreatic enzyme dose used in the sample was 7126.24 (3860 – 11,905) IU/kg.

**Table 3:** Most frequent clinical conditions and abdominal symptoms reported in the questionnaire and their relationship with FC levels.

Pain	FC median (range)	p^a^
Yes (*n* = 8)	148 (30–1000)	0.96
No (*n* = 11)	155 (30–961)	
**Flatulence**		
Yes (*n* = 11)	259 (30–1000)	0.11
No (*n* = 8)	104.5 (30–342)	
**Pulmonary exacerbation**		
Yes (*n* = 8)	583 (30–1000)	0.24
No (*n* = 14)	165 (30–961)	
**Antibiotic use**^b^		
Yes (*n* = 3)	155 (70–259)	0.47
No (*n* = 19)	207 (30–1000)	
**Inhaled antibiotic use**		
Yes (*n* = 10)	190 (30–1000)	0.51
No (*n* = 12)	191 (30–961)	
**Colonization by *P. aeruginosa***		
Yes (*n* = 9)	85 (30–1000)	0.12
No (*n* = 13)	259 (30–961)	
**Pancreatic insufficiency**		
Yes (*n* = 18)	279 (30–1000)	0.10
No (*n* = 4)	82 (70–175)	
**Male sex**		
Yes (*n* = 13)	175 (30–961)	0.38
No (*n* = 9)	342 (78–1000)	
**Low BMI**		
Yes (*n* = 4)	103 (70–961)	0.55
No (*n* = 18)	233 (30–1000)	
**F508del in homozygosity**		
Yes (*n* = 5)	175 (30–961)	0.90
No (*n* = 17)	342 (78–1000)	

a Mann-Whitney.

b In therapy for acute infectious condition other than pulmonary exacerbation.

## Discussion

The measurement of fecal calprotectin in patients with CF indicated values related to intestinal inflammation in almost all cases. This was the most relevant finding of the present assessment. A total of 86.3% of patients showed FC levels above 50 mg/g, the cut-off value suggested by the manufacturer to increase sensitivity for intestinal inflammation, and the range most used in existing studies. Additionally, based on quantitative evaluation of FC in people with CF and controls, Jaudszus et al. [13] established a cut-off value of 44.1 mg/g to distinguish between the two groups, similar to the 50 mg/g used to indicate the presence of intestinal inflammation.

In healthy children, FC levels are higher in early months but decrease with age as intestinal immunity matures. Exposure to the intestinal microbiota, which begins to establish after leaving the uterus, and exposure to food antigens in a context where the intestinal mucosa functions as an immature barrier, promote the migration of granulocytes and macrophages rich in FC to the intestinal epithelium. From age 4 onwards, FC levels in healthy children begin to approach the adult normal range more rapidly, still maintaining the inverse correlation between age and FC level [14]. Even considering that the sample in the present study is pediatric, the increase in FC that can be attributed to immature immunity in children is quite discreet after age 4 and significantly lower than the results obtained, even among those without GI symptoms.

The finding of elevated FC levels in people with CF is consistent with related studies and with the theoretical framework proposed to explain intestinal inflammation caused by the disease.

Dysfunctional CFTR directly contributes to the genesis of intestinal inflammation by stimulating pro-inflammatory pathways in the intestinal inflammatory response [15,16]. and is associated with reduced efficacy of the innate immune response (low levels of beta-defensin 2, an antimicrobial protein), which can trigger a dysregulated immune response and a pro-inflammatory state [17]. Endoscopic evaluations have identified high concentrations of inflammatory interleukins and visualized mucosal changes throughout the GI tract, such as edema, enanthema, and ulcers [9]. Finally, the well-known intestinal dysbiosis in CF is characterized by a predominance of pro-inflammatory microorganisms, and the intestinal mucosa shows increased permeability due to changes in tight junctions, allowing the translocation of microorganisms and pro-inflammatory substances [16].

Several studies have sought associations between FC levels in people with CF and clinical manifestations. The evaluations show highly variable results. Rumman et al. [18]. found a reduction in fecal calprotectin levels associated with the use of inhaled antibiotics. Adriaanse et al. [19]. and Garg et al. [20] observed a negative correlation between age and calprotectin level. Parisi et al. [21] and Imanzadeh et al. [22] found a negative correlation between lung function and the presence of pulmonary exacerbation with FC levels. Schnapp et al. [23] and Imanzadeh et al. [22] found an association between systemic antibiotic therapy and reduced FC levels. Dhaliwal et al. [24] identified an inverse correlation between weight and height Z-scores and FC levels. Finally, Werlin et al. [25] identified a relationship between exocrine pancreatic insufficiency and higher FC levels.

The present research did not find an association or correlation between symptoms or clinical characteristics and FC levels. The analysis of the relationship between FC levels and the clinical features of flatulence, pulmonary exacerbation, colonization by P. aeruginosa, and pancreatic insufficiency nearly reached statistical significance, which would be biologically plausible and aligns with findings from other studies [11]. Frequent pulmonary exacerbations and colonization by P. aeruginosa are well-established associations in cystic fibrosis. The large amounts of airway secretions produced during these exacerbations, when swallowed, can raise fecal calprotectin levels simply because the calprotectin present in airway secretions reaches the colon [21]. The absence of associations or correlations may arise from a limitation of this study, namely, the reduced number of participants. Almost all the cited studies included more than 30 participants, which confers greater power in observing statistical significance and allows for more consistent conclusions.

It is important to highlight the reasons why the present sample size was so small. The main reason was funding for FC quantification tests, which are not part of the laboratory exams offered by the institution. The authors had to stop including participants in the study once they reached the number of tests available. Other, less significant factors included participants dropping out due to the inconvenience of collecting, storing, and transporting stool samples, improper home storage, or personal reasons. Such difficulties, especially in places with limited funding for science, reinforce the importance of seeking partnerships to organize multicenter studies and obtain more precise and conclusive results.

The most common abdominal symptoms were abdominal pain and flatulence, with each affecting about half of the participants. Pain is a recurrent symptom that became more noticeable after control of more severe morbidities in these patients. Studies indicate a high prevalence of this complaint in children with CF, with abdominal pain being the main manifestation in most cases, reported by up to 60% of patients [8]. In Brazil, there are no records of systematic studies on gastrointestinal symptoms in people with CF.

The evaluation and management of pain in children with CF are essential since painful symptoms may limit the patient’s participation in disease-related care and negatively impact their well-being and quality of life [26]. Additionally, abdominal symptoms in general are associated with mental health disorders in adolescents and adults with CF [27].

Besides the gastrointestinal comorbidities known to be associated with CF and intestinal inflammation—which did not show an association with symptoms in this study—other mechanisms may justify such manifestations in people with CF. Dysfunctional CFTR is expressed in neurons of the enteric nervous system, responsible for mediating vascularization, intestinal motility, secretion and absorption of substances, and intestinal inflammation, possibly contributing to the onset of gastrointestinal symptoms [28].

In adults with CF, functional GI disorders are more prevalent [29]. In the pediatric population, Smith et al. evaluated the prevalence of functional disorders described by ROME VI criteria in people aged 0 to 18 years, finding a statistically significant association between FC and a higher occurrence of functional abdominal pain diagnosis [30]. Disorders of the brain-gut axis are therefore also causes of abdominal symptoms in these individuals and may be associated with the abdominal pain reported by study participants.

This study demonstrates a high prevalence of intestinal inflammation measured by FC levels in children with CF at a Brazilian reference center for disease treatment. Despite the frequent occurrence of abdominal symptoms, it was not possible to establish a significant association between FC levels and clinical manifestations or gastrointestinal symptoms. The small sample size makes it hard to analyze a multifactorial outcome such as GI symptoms. These findings contribute to the understanding of gastrointestinal manifestations in CF patients. The authors highlight the importance of systematic investigation of these symptoms to better understand the impact of CF on pediatric patients. Therapeutic strategies that manage to mitigate such manifestations would have high potential to improve their quality of life.

## Funding

This work was supported by the State University of Campinas Teaching Research and Extension Support Fund (FAEPEX), and JDR receives CNPq Research Productivity Grant (#300024/2025-8) - CNPq: Conselho Nacional de Desenvolvimento Científico e Tecnológico (Brazilian National Council for Scientific and Technological Development).

## Data availability statement

The data that support the findings of this study are available from the corresponding author.

## Conflicts of interest

The authors declare that they have no known competing financial interests or personal relationships that could have appeared to influence the work reported in this paper.
